# From exchange to action: how hospitals receive and use health-related social needs data from external sources

**DOI:** 10.1093/jamia/ocag092

**Published:** 2026-06-11

**Authors:** Chelsea Richwine, Wei Chang, Bradley Iott

**Affiliations:** Office of the National Coordinator for Health Information Technology, United States Department of Health and Human Services, Washington, DC, United States; Office of the National Coordinator for Health Information Technology, United States Department of Health and Human Services, Washington, DC, United States; Division of General Medicine, Department of Internal Medicine, University of Michigan Medical School, Ann Arbor, MI, United States

**Keywords:** Health-related social needs, social determinants of health, interoperability, health information technology, health information exchange, data sharing

## Abstract

**Objective:**

To understand how hospitals receive and use data on patients’ health-related social needs (HRSN) from external sources, and how methods of receipt relate to use.

**Materials and methods:**

Using 2024 nationally representative survey data on hospitals (*N *= 2146) we described external sources of receiving HRSN data and variation by hospital characteristics, and how methods of receipt relate to uses of data to support patient care.

**Results:**

In 2024, 70% of hospitals received HRSN data from external sources—via health information exchanges (HIEs) (51%), electronic health record (EHR) vendor networks (40%), national networks (36%), referral platforms (28%), other healthcare organizations (24%) and community-based organizations (20%). Large, urban, system-affiliated, non-profit and government-owned hospitals, value-based care participants, and those with an Epic EHR had higher rates of receiving HRSN data from any source compared to their counterparts. About half of hospitals reported using data received for various purposes (e.g., referrals, discharge planning). Hospitals that received data via HIEs or EHR vendor networks or directly from other organizations were more likely to use data across measured uses. Hospitals that received data via referral platform were more likely to use data for referrals, informing community needs, and population health analytics.

**Discussion:**

Many hospitals receive HRSN data from external sources, often through existing connections to health information networks, which was associated with greater internal and downstream use.

**Conclusion:**

Efforts to improve bi-directional exchange between health and social service organizations may enable hospitals to more effectively identify and address patients’ HRSN.

## Introduction

Hospitals play an important role in addressing patients’ health-related social needs (HRSN)—social and economic needs such as access to food, housing, and reliable transportation—that impact health and well-being.^1^^,^^2^ Unaddressed HRSNs increase the risk of chronic disease and adverse health outcomes, leading to increased medical costs and avoidable emergency department use.^3^ National social care standards increasingly drive hospitals to identify and address HRSNs,^4^ which is critical to promoting whole-person health and reducing healthcare costs.^5^^,^^6^ As of 2023, 88% of hospitals collected HRSN data locally through screening, with 72% documenting data in a structured format (i.e., via structured electronic screening tool mapped to standards like LOINC and SNOMED CT, or diagnosis codes, such as ICD-10 Z codes) and 16% using unstructured methods like narrative, free-text notes.^7^ Hospitals also receive HRSN data in these different formats from external sources, including directly from other healthcare organizations and community/social service organizations, or via intermediaries such as community referral platforms and broader health information networks such as state, regional, local health information exchanges (HIEs), national networks that connect providers, payers and other entities (e.g., Carequality, eHealth Exchange, and CommonWell Health Alliance), and electronic health record (EHR) vendor networks.^8–10^

While hospitals receive HRSN data from various sources, several challenges—such as limited technical capabilities or community resources—constrain the flow and utility of this information.^11^ Although community-based referral platforms can facilitate HRSN data exchange and care coordination, hospital adoption remains low.^12^^,^^13^ Additionally, interoperability challenges between the healthcare and social care sectors—including terminology differences and low standards adoption—hinder data sharing.^11^^,^^14^ Community efforts are underway to address these standards challenges, including the Gravity Project, which develops consensus-based data standards to improve HRSN data capture and exchange, and the DirectTrust Information Exchange for Human Services (IX4HS) project.^11^^,^^15^^,^^16^ Standardized HRSN data can be more easily integrated into EHRs to support prompting or pre-populating of screening and can be more easily exchanged with other organizations.^17–19^

While most research has focused on hospitals’ collection and documentation of HRSN data,^7^^,^^20–24^ externally received HRSN data—such as patient-level screening data from other organizations—can help hospitals more comprehensively understand patients’ needs and deliver better care. In 2022, 61.4% of hospitals received HRSN data from external sources, with higher likelihood among hospitals participating in value-based payment programs and those using Epic.^8^ However, little is known about the sources or methods through which HRSN data are received or how these data are used.

This study examines *how* hospitals receive HRSN data—via intermediaries and directly from other healthcare and community/social service organizations—and whether specific sources or methods are associated with greater use of data for different purposes. Identifying common receiving methods and their relationship to use can illuminate the efficacy of different approaches for supplementing hospitals’ own HRSN collection, and help reduce burden associated with duplicative data collection.^8^ Understanding how hospital characteristics are associated with different sources and use of data can highlight opportunities to improve hospitals’ ability to identify and address HRSN.

## Methods

This cross-sectional study did not require ethics committee approval because it did not involve human participants. We followed the Strengthening the Reporting of Observational Studies in Epidemiology (STROBE) reporting guideline for cross-sectional studies.

### Data collection and study population

Data came from the 2024 Information Technology (IT) Supplement to the American Hospital Association (AHA) Annual Survey, a nationally representative survey of U.S. hospitals. The IT Supplement was sent to chief executive officers of all U.S. hospitals with instructions that the individual most knowledgeable about health IT at their hospital should complete the survey. The IT Supplement was fielded April to September 2024 and 2260 nonfederal acute care hospitals responded (52% response rate).

Our analytic sample was limited to nonfederal acute care hospitals that used an EHR and had complete information for key outcomes and characteristics (*N* = 2146). Certain types of hospitals (small, rural, independent, government-owned hospitals) were less likely to respond to the IT Supplement, possibly due to resource constraints. To help adjust for differences in response, all estimates were weighted to the population of hospitals represented in the AHA Annual Survey. Hospital-level weights were derived by the inverse of the predicted propensity to respond to the IT supplement. Response was predicted using a logistic regression model, including size, ownership, teaching status, system membership, cardiac intensive care unit, urban status, and region.

Social deprivation index measures came from the Robert Graham Center using 2015–2019 American Community Survey data.^25^ Medicaid discharges and uncompensated burden data came from 2022 Healthcare Provider Cost Reporting Information System Public Use File, which contains annual cost reports submitted by Medicare-certified institutional providers.^26^

### Outcomes and measures

#### Receipt of HRSN data

Independent variables indicate the sources from which hospitals received HRSN data from outside their hospital/health system including state, regional, and/or local HIEs, EHR vendor-based networks (e.g., Epic’s Care Everywhere), national networks, social service or community-based referral platforms (e.g., Unite Us, FindHelp), other healthcare organizations, or community/social service organizations.^27–29^ All measures were defined as binary variables indicating hospitals that reported receiving data from a given source. Measures were not mutually exclusive; hospitals may receive data from more than one source. We also defined a binary variable indicating the share of hospitals that received data from at least one external source.

#### Use of HRSN data

Outcomes included hospital-reported uses of HRSN data, including for making referrals to social service organizations, clinical decision making, discharge planning, population health analytics, informing community needs assessment, and supplementing screening data collected internally (e.g., to prompt screening, prepopulate screening tools).

To understand the extent to which hospitals relied on data collected internally versus from external sources for each use, we defined six categorical variables indicating hospitals’ use of HRSN data both collected and received, received from external sources only, collected internally only, or not used. To capture hospitals’ use of data received from external sources, we also defined six binary variables indicating hospitals’ use of data received for each measured use. See Appendix Tables A1 and A2, available as supplementary material  *Journal of the American Medical Informatics Association* online for survey questions used to create key measures.

#### Covariates

We included several hospital characteristics that may influence hospitals’ ability to receive and use HRSN data, including hospital size (small, <100 beds; medium, 100–399 beds; large, >400 beds), critical access status, system membership, rural location, hospital ownership (for-profit, nonprofit, government), participation in value-based care (i.e., accountable care organization, patient-centered medical home program, or pay-for-performance arrangement). We also examined hospitals using an Epic EHR versus all other vendors since Epic accounts for over half of the market share for nonfederal acute hospitals, and has been shown to be associated with greater receipt of HRSN data.^8^ To identify safety-net hospitals that provide care for individuals regardless of their insurance status or ability to pay, we used a composite variable that captured whether a hospital was in the top 10% of: Medicaid discharges within their state, uncompensated burden within their state, or their service area social deprivation index—a measure of area-level deprivation derived from demographic and household characteristics which has been shown to be associated with hospital engagement in interoperable exchange.^25^^,^^30^

### Statistical analysis

First, we described our analytic sample of all hospitals and among those that received external HRSN data. We then described how hospitals received HRSN data from external sources and how sources of receipt varied by hospital characteristics. Differences were assessed using *χ*^2^ tests of independence.

Next, we described hospitals’ use of HRSN data (both collected internally and received from outside sources) for common uses including making referrals, clinical decision making, discharge planning, population health analytics, informing community needs assessments, and supplementing screening data collected internally (for data received only).

Finally, to understand how methods of receiving data relate to hospitals’ use of externally received HRSN data, we implemented six weighted logistic regressions predicting hospitals’ use of HRSN data based on the source(s) of HRSN data received and other hospital and area characteristics. Relationships were reported in terms of average marginal effects (AME) which allow us to describe the average effects of different methods of receiving data on the likelihood that a hospital used data for a specific purpose.

All models controlled for hospital characteristics described above. Given that most hospitals that received HRSN data through an EHR vendor network were Epic users (88%), EHR vendor was not included in regression models since these variables are highly correlated. For all models, *P* value <.05 were considered statistically significant. Data analyses were performed between May to July 2025 using Stata/SE, version 17.0 (StataCorp).

## Results

The total study population included 2146 hospitals (N [Weighted %]), with 1562 (70%) reporting receiving HRSN data (Table 1). The characteristics of those hospitals which received HRSN data were similar to the characteristics of all hospitals in the sample. Among those who received HRSN data, 721 (51%) were small, 612 (37%) were medium, and 229 (13%) were large hospitals. Hospitals receiving HRSN data were more likely to participate in value-based care initiatives (65% vs 57%), to be nonprofit (75% vs 67%), and to have an Epic EHR (61% vs 52%).

**Table 1.** Sample characteristics, overall and by receipt of HRSN data from at least one external source.

	All hospitals (*N* = 2146)		Receive HRSN (*N* = 1562)	
	*N*	Weighted %	*N*	Weighted %
**Collect HRSN data**				
** Yes**	2036	94%	1516	97%
** No or don’t know**	110	6%	46	3%
**Receive HRSN data**				
** Yes**	1562	70%	1562	100%
** No or don’t know**	584	30%	–	–
**Bed Size**				
** Small (<100 beds)**	1003	51%	721	51%
** Medium (100–399 beds)**	852	38%	612	37%
** Large (>400 beds)**	291	11%	229	13%
**Location**				
** Rural**	433	22%	296	21%
** Urban**	1713	78%	1266	79%
**Region**				
** Northeast**	245	12%	191	13%
** Midwest**	714	30%	535	31%
** South**	799	38%	539	35%
** West**	388	20%	297	21%
**Critical access hospital**				
** Yes**	596	31%	432	31%
** No**	1550	69%	1130	69%
**System affiliation**				
** Independent**	490	31%	319	28%
** System affiliated**	1656	69%	1243	72%
**Ownership**				
** For-profit**	231	13%	72	6%
** Non-profit**	1547	67%	1245	75%
** Government**	368	20%	245	19%
**Value-based care**				
** Yes**	1301	57%	1054	65%
** No**	845	43%	508	35%
**Safety net hospital**				
** Yes**	542	26%	407	27%
** No**	1604	74%	1155	73%
**EHR vendor**				
** Market leader (Epic)**	1202	52%	115	61%
** All other vendors**	934	48%	547	39%

### External sources of HSRN data

**Figure 1.**
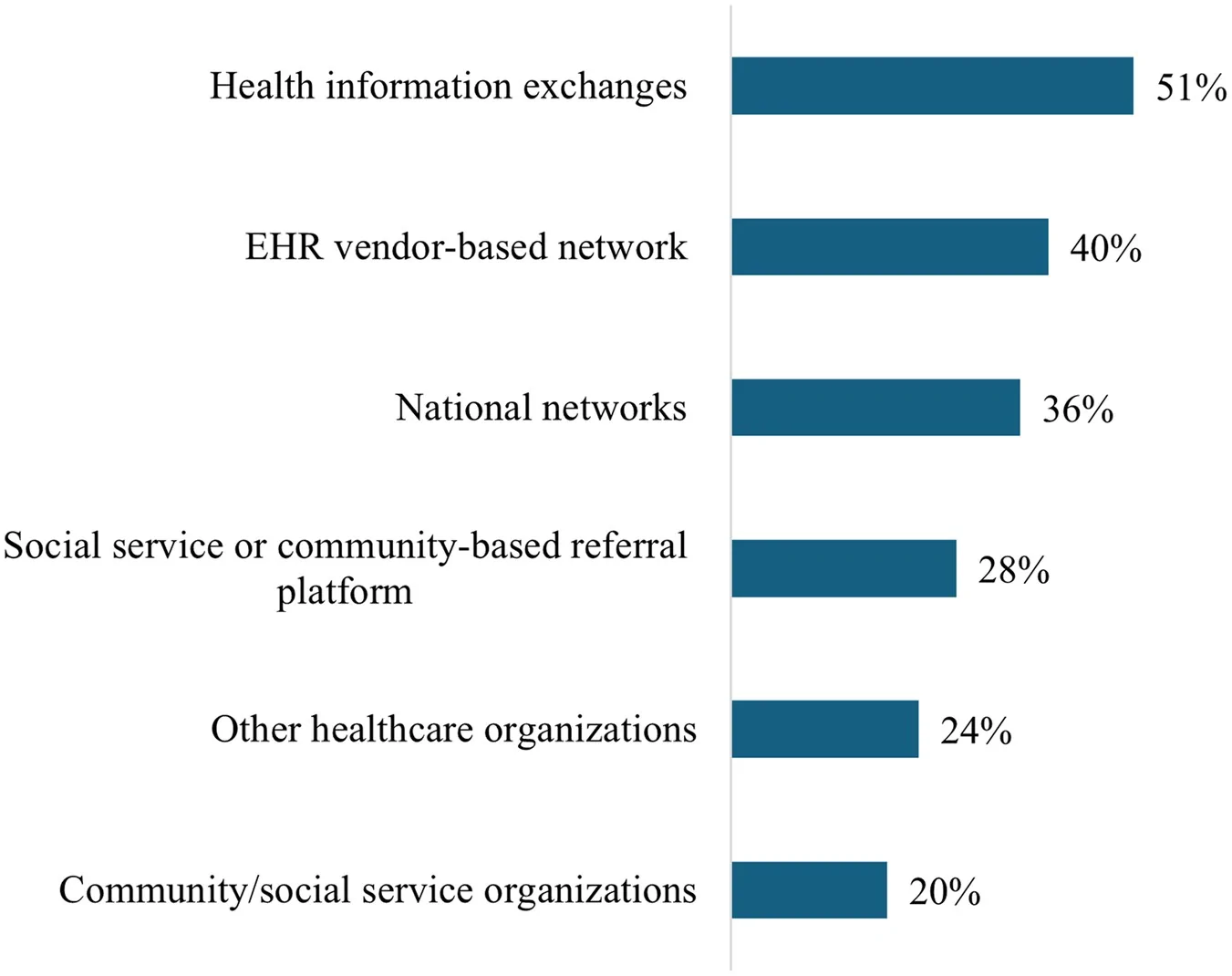
Sources from which hospitals receive data on patients’ health-related social needs outside their hospital/health system, 2024. *Notes*. The denominator includes all hospitals in the analytic sample (*N* = 2146).

Figure 1 shows the sources from which hospitals received HRSN data from outside their hospital/health system. The most common source of HRSN data was from HIEs (51%), followed by EHR vendor-based networks (40%), national networks (36%), social service or community-based referral platforms (28%), other healthcare organizations (24%), and community/social service organizations (20%).

### Hospital characteristics associated with HRSN data sources

Table 2 shows the hospital and area characteristics associated with receipt of HRSN data from outside sources. Consistent with prior literature, large, urban, system-affiliated, nonprofit and government-owned hospitals, value-based care participants and those using an Epic EHR had significantly higher rates of receiving HRSN data from any source, and across specific sources of HRSN data, compared to their counterparts. Epic users were substantially more likely to receive HRSN data through their vendor-provided network (68% vs 10% for other vendors). Critical access hospitals had lower rates of receiving data from national networks, EHR vendor networks, and community-based referral platforms compared to non-critical access hospitals. We observed no significant differences in methods of receiving HRSN by safety net status.

**Table 2.** Methods of receiving HRSN data from outside sources, by hospital characteristics

		Sources of HRSN Data					
Hospital Characteristics	Any Source	HIEs	EHR Vendor Network	National Network	SS/CBRP	Other HCOs	C/SS Orgs
Bed Size							
Small (<100 beds) (ref)	70%	49%	35%	32%	23%	23%	19%
Medium (100–399)	69%	51%	42%*	38%*	32%*	22%	19%
Large (>400)	77%*	57%*	53%*	49%*	41%*	32%*	25%*
Location							
Rural	66%	50%	30%	30%	19%	20%	16%
Urban	72%*	51%	43%*	38%*	31%*	25%*	21%*
Region							
Northeast (ref)	76%	60%	50%	50%	33%	32%	23%
Midwest	73%	51%*	37%*	34%*	29%	29%	29%*
South	65%*	47%*	40%*	36%*	25%*	18%*	11%*
West	74%	53%	39%*	33%*	31%	21%*	19%
Critical access status							
CAH	71%	52%	33%	32%	20%	22%	19%
Non-CAH	70%	50%	43%*	38%*	32%*	24%	20%
System affiliation							
Independent	64%	46%	27%	31%	10%	20%	12%
System-affiliated	73%*	53%*	46%*	39%*	37%*	25%*	23%*
Ownership							
For-profit (ref)	32%	21%	18%	12%	5%	4%	3%
Non-profit	80%*	58%*	47%*	44%*	38%*	28%*	25%*
Government	65%*	45%*	30%*	27%*	12%*	22%*	14%*
Value-based care							
Yes	80%	56%	50%	42%	37%	28%	28%
No	58%*	43%*	27%*	29%*	18%*	17%*	9%*
Safety net hospital							
Yes	73%	54%	39%	35%	29%	23%	18%
No	70%	50%	40%	37%	28%	24%	20%
EHR vendor							
Marked leader (Epic)	83%	62%	68%	47%	39%	31%	22%
All other vendors	57%*	39%*	10%*	25%*	17%*	16%*	17%*

*Notes*. The denominator includes all hospitals in the analytic sample (*N *= 2146). HIEs, Health Information Exchanges; SS/CBRP, Social service or community-based referral platform; Other HCOs, Other healthcare organizations; C/SS orgs, Community/social service organizations.

* Indicates statistically significant difference from reference category (*P* < .05).

**Figure 2.**
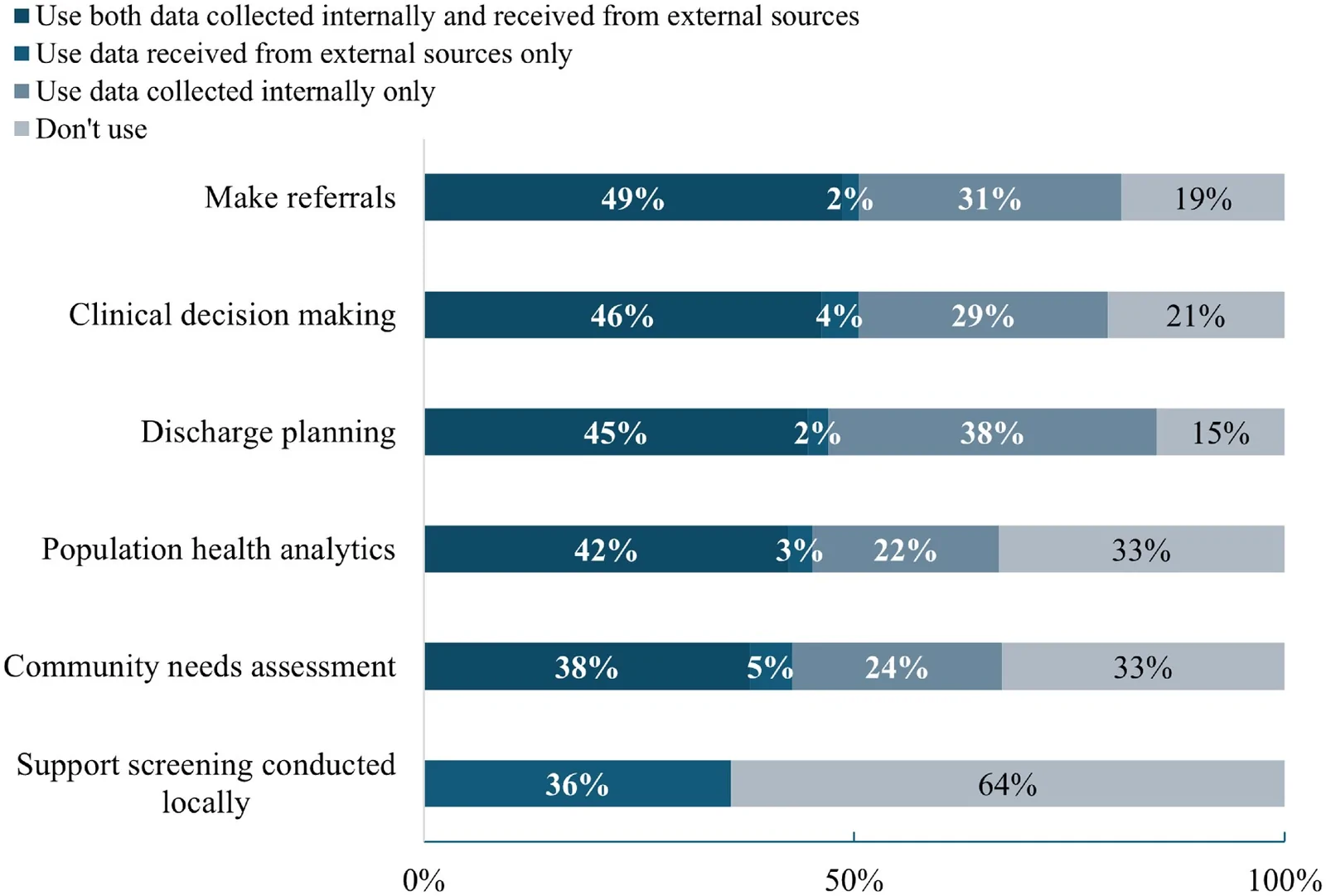
Hospitals’ use of data collected internally or received from external sources for different purposes. *Notes*. The denominator includes all hospitals in the analytic sample (*N* = 2146).

Figure 2 shows the different uses of HRSN data and the extent to which hospitals relied on data collected internally versus received from external sources for each use. The most common uses of HRSN data overall were for discharge planning (85%), making referrals (81%), and clinical decision-making (79%); while about two-in-three hospitals (67%) used data for population health analytics and informing community needs assessments (sum of first three categories in Figure 2, see Appendix Table A3, available as supplementary material  *Journal of the American Medical Informatics Association* online). About one-in-three hospitals (36%) used data received from external sources to support screening locally.

Across all common use cases, the largest group of hospitals used both data collected internally and received from external sources, while a smaller group used data collected internally only. About half of all hospitals used data collected and received for making referrals (49%), clinical decision-making (46%), and discharge planning (45%) compared to about one-third of hospitals (29%-38%) that used data collected internally only for these purposes. Hospitals also relied more on data collected and received for population health analytics (42%) and informing community needs assessments (38%) than on data collected internally only (22% and 24%, respectively). Hospitals that relied only on HRSN data received from external sources were the smallest group for each use case, ranging from 2% of hospitals for making referrals and discharge planning to 5% of hospitals for informing community needs assessments.

### Associations between use of HRSN data and external source

Table 3 displays associations between hospitals’ receipt of HRSN data from external sources and specific uses of data received (full output in Appendix Table A4, available as supplementary material  *Journal of the American Medical Informatics Association* online). Hospitals that received HRSN data through an HIE or EHR vendor network were significantly more likely to use HRSN data for five of six use cases compared to hospitals that received HRSN data from other external sources. For example, hospitals that received HRSN data through their HIE were about 18 percentage-points more likely to use data to support screening conducted internally (95% CI 0.123–0.234) and 21 percentage-points more likely to use data for population health analytics (95% CI 0.162–0.263). Hospitals that received HRSN data through a national network were also more likely to use HRSN data to support screening (AME = 0.177, 95% CI 0.123–0.231), for population health analytics (AME = 0.049, 95% CI 0.000–0.098), and to inform discharge planning (AME = 0.077, 95% CI 0.026–0.127).

**Table 3.** Relationship between source(s) and use of HRSN data received from other hospitals/health systems

Panel A:	(1) Support screening		(2) Make referrals		(3) Population health	
VARIABLES	AME	(95% CI)	AME	(95% CI)	AME	(95% CI)
**Sources of HRSN Data**						
** HIEs**	0.178**	(0.123–0.234)	0.066**	(0.016–0.116)	0.212**	(0.162–0.263)
** EHR vendor network**	0.134**	(0.082–0.186)	0.061**	(0.015–0.108)	0.085**	(0.038–0.132)
** National network**	0.177**	(0.123–0.231)	0.045^+^	(−0.002–0.092)	0.049*	(0.000–0.098)
** SS/CBRP**	−0.015	(−0.068–0.039)	0.083**	(0.037–0.130)	0.148**	(0.099–0.198)
** Other HCOs**	0.109**	(0.047–0.171)	0.160**	(0.111–0.208)	0.114**	(0.060–0.169)
** C/SS orgs**	−0.074*	(−0.135–−0.014)	0.092**	(0.040–0.144)	0.067*	(0.014–0.120)
**Observations**	1562		1562		1562	

Panel B	(4) Community needs assessment		(5) Clinical decision-making		(7) Discharge planning	
VARIABLES	AME	(95% CI)	AME	(95% CI)	AME	(95% CI)
**Sources of HRSN Data**						
** HIEs**	0.145**	(0.091–0.199)	0.139**	(0.085–0.193)	0.028	(−0.025–0.080)
** EHR vendor network**	0.040	(−0.009–0.089)	0.079**	(0.031–0.128)	0.057*	(0.007–0.108)
** National network**	0.023	(−0.028–0.074)	0.036	(−0.013–0.085)	0.077**	(0.026–0.127)
** SS/CBRP**	0.227**	(0.175–0.279)	−0.031	(−0.081–0.018)	0.004	(−0.048–0.055)
** Other HCOs**	0.121**	(0.064–0.177)	0.154**	(0.104–0.204)	0.196**	(0.145–0.247)
** C/SS orgs**	0.083**	(0.027–0.139)	0.103**	(0.050–0.156)	0.096**	(0.038–0.155)
**Observations**	1562		1562		1562	

*Notes*. Denominator includes hospitals that received HRSN data from at least one external source (*N *= 1562). Each column in Table 3 reports output from a separate weighted logistic regression and control for hospital size, location, ownership, critical access status, and participation in value-based care. See Appendix Table A4, available as supplementary material  *Journal of the American Medical Informatics Association* online for full output. All coefficients are reported in terms of average marginal effects (AME). 95% Confidence Intervals (CI) are in parentheses. Acronyms used for sources of HRSN data include: HIEs, Health Information Exchanges; EHR, Electronic Health Record; SS/CBRP, Social service or community-based referral platform; Other HCOs, Other healthcare organizations; C/SS orgs, Community/social service organizations. Asterisks indicate statistically significant difference from reference category:

**P < .01,

*P < .05.

^+^Indicates marginal significance: *P* < .10.

Hospitals that received HRSN data through a community-based referral platform were significantly more likely to use HRSN data for making referrals (AME = 0.083, 95% CI 0.037–0.130), population health analytics (AME = 0.148, 95% CI 0.099–0.198), and to inform community needs assessments (AME =0.227, 95% CI 0.175–0.279). Hospitals that received data from other healthcare organizations were significantly more likely to use data for all purposes compared to hospitals that received data from other external sources. The largest effects were observed for discharge planning (AME = 0.196, 95% CI 0.145–0.247), clinical decision-making (AME = 0.154, 95% CI 0.104–0.204), and making referrals (AME =0.160, 95% CI 0.111–0.208). Hospitals that received data from community/social service organizations were significantly more likely to use data for all purposes except to support screening (AME = −0.074, 95% CI −0.135 to −0.014). Hospitals that received data from community/social service organizations were about 10 percentage-points more likely to use data for clinical decision-making (95% CI 0.050–0.156), discharge planning (95% CI 0.038–0.155), and making referrals (95% CI 0.040–0.144).

## Discussion

Recent studies have documented the rise in the collection and receipt of HRSN data by healthcare organizations.^20^^,^^31^ Building on this work, our findings indicate 70% of hospitals received HRSN data from external organizations in 2024, up from 61.4% in 2022.^8^ This increase may reflect greater participation in health information networks or broader engagement in interoperable exchange, which has been shown to be a significant predictor of receiving HRSN data from external sources.^8^^,^^32^

Hospitals typically receive HRSN data through existing health information networks, most commonly through HIEs, but also EHR vendor networks and broader national networks, designed for clinical data exchange. This may be due to the ease of exchanging data via existing infrastructure, especially when vendor-provided tools are already integrated into existing workflows.^9^ Hospitals less frequently received HRSN data from community-based referral platforms and community/social service organizations, which may be partially explained by implementation barriers that slow the adoption of these technologies and hinder interoperable exchange.^13^ Taken together, our findings suggest that fostering these existing connections and working to alleviate interoperability barriers may help providers have timely and actionable information needed to address patients’ needs and improve healthcare quality.

Consistent with prior literature, large, urban, system-affiliated hospitals were more likely to receive HRSN data than small, rural, independent organizations, likely due to greater resources or advanced EHR capabilities.^32^ Hospitals using an Epic EHR had a higher likelihood of receiving data overall than other vendors, likely driven by Care Everywhere, which enables exchange between Epic users. Value-based care participants also had a higher likelihood of receiving data from all sources, paralleling findings observed for HRSN data collection,^7^ suggesting greater incentive to identify and address HRSN, as these programs tie community needs assessments and population health improvement to payment. Despite serving patients with high social risk, safety-net hospitals were equally likely to receive external HRSN data, though they may document HRSN while being less likely to engage in interoperable exchange.^30^ Rural and critical access hospitals were also less likely to receive HRSN data, consistent with prior work showing these facilities less commonly collect HRSN or offer resources to address patients’ social needs, suggesting a need to determine how to support rural and critical access hospitals in obtaining and using HRSN data.^7^^,^^33^

About half of all hospitals used external HRSN data—alone or with internal data—to inform clinical decisions and discharge planning, make referrals, population health analytics, and community needs assessments. One-in-three hospitals used data received to prompt or pre-populate screening, indicating the utility of external HRSN data for patient care and population health. Prior work indicates nearly all hospitals screen for HRSN and use data collected for different purposes.^7^ Our findings indicate less than 1-in-3 hospitals relied only on data collected internally for these uses—possibly due to a perception that locally collected data is more actionable, and possibly more reliable, than external sources. Greater adoption of standards to identify HRSN problems and interventions may increase reliability and exchangeability.

While many hospitals used HRSN data for different purposes, we observed variation in use by source. Hospitals receiving HRSN data from clinical sources were more likely to use it to prompt social risk screening than facilities receiving HRSN data from community-based referral platforms or community/social service organizations, likely reflecting low referral platform adoption and comparative ease of using existing HIE infrastructure. In addition to reducing the need for making additional technological connections, HRSN data from other clinical organizations may reflect screening conducted at those facilities, enabling pre-population of local screening tools or prompting local screening. Similarly, HRSN data from community-based referral platforms may be most useful for tracking referral status or informing population health initiatives.^12^

Receiving data from HIEs was associated with a greater likelihood of using data for community needs assessments or population health analytics compared to those receiving data from national networks. This highlights the value of HIEs in facilitating the exchange and use of local HRSN data that are particularly useful for informing community/population health initiatives, especially when those HIEs have a care coordination or closed-loop referral platform.^19^ While national networks offer broad coverage for clinical data exchange, less HRSN data may flow through them less frequently; and data elements exchanged at a national scale may be less actionable than local sources.

Despite policy to increase HRSN collection, more work is needed to design tools that facilitate bi-directional exchange between health and social service organizations,^34^ including connecting healthcare organizations to existing platforms and leveraging HIEs and national networks to serve as HRSN data hubs. The Trusted Exchange Framework and Common Agreement™ (TEFCA™) may play a role as HRSN data increasingly flows through clinical sources. On the social care side, community and social service organizations need sufficient technical infrastructure and resources to capture and exchange HRSN information with healthcare organizations. Greater standardization and common data collection across health and community/social service organizations may improve cross-organization data sharing, and make it easier to leverage existing technology to exchange and use information to improve patient and population health.^11^

### Limitations

Hospitals self-reported sources and uses of externally received HRSN data. We did not capture the volume or frequency of HRSN data received, nor what circumstances triggered external HRSN data pulls. We were unable to measure the content nor volume of received HRSN data from specific sources (e.g., if 90% of data is received via HIE and 10% from social services organizations directly). Measuring when, how often, and which HRSN data are exchanged may clarify the current landscape of HRSN exchange. Finally, engagement in interoperable exchange was removed from the survey in 2024, so we were unable to include this measure as a control as prior work has done.^8^

## Conclusion

In 2024, most hospitals received HRSN data from external sources and many did so through existing connections to health information networks and less commonly through a community-based referral platform or directly from other health and community/social service organizations. About half of hospitals used a combination of data collected internally and received from external sources to provide or coordinate care for their patients, indicating the utility of data received from external sources. Hospitals’ receipt of data through HIEs, EHR vendor networks, and directly from other healthcare organizations were most consistently associated with greater use of data received for different purposes. While there are several efforts aimed at increasing the collection of HRSN data, efforts to improve bi-directional exchange between health and social service organizations would enable hospitals to more effectively identify and address HRSN, which is critical to promoting whole-person health and reducing healthcare costs.

## Supplementary Material

ocag092_Supplementary_Data

## Data Availability

Data are available for purchase from the American Hospital Association: https://www.ahadata.com/aha-healthcare-it-database.
