# Supplementary material for: From exchange to action: how hospitals receive and use health-related social needs data from external sources
Source: J Am Med Inform Assoc. 2026 Jun 11;33(9):1627–35. doi: 10.1093/jamia/ocag092 (PMC13580729; doi:10.1093/jamia/ocag092)
Supplement: Supplementary file 1 [file ocag092_supplementary_data.docx]

**APPENDIX**

Table A1. Sources from which HRSN are Received Survey Question

From which sources outside your hospital/health system does your hospital electronically receive data on patients’ social needs? (Check all that apply)

a. Health information exchanges (i.e. state, regional, and/or local HIE/HIO(s))

b. Social service or community-based referral platform (such as Unite Us or Aunt Bertha)

c. National Networks (e.g. CommonWell, eHealthExchange, Carequality)

d. EHR vendor-based network (e.g. Epic's Care Everywhere)

e. Other healthcare organizations

f. Community/social service organizations

g. Other (electronically receive social needs data)

Other description (electronically receive social needs data, free text)

h. Do not receive data on health-related social needs from any external sources (go to 10)

i. Do not know (electronically receive social needs data) (go to 10)

Table A2. Uses of Externally Sourced HRSN Data Survey Question

If you indicated receiving data on patients' health related social needs from sources outside your hospital/health system, how do you use these data? (Check all that apply)

a. For screening at our hospital (e.g., prompt screening, prepopulate screening tool)

b. For referrals to social service organization

c. For population health analytics

d. To inform community needs assessment or other equity initiatives

e. To inform clinical decision making

f. To inform discharge planning

g. Other (using external data)

Other description (using external data)

Table A3. Hospitals’ use of HRSN data collected or received.

| Uses of HRSN data: | Use data collected or received | Use data received | Use data collected |
| --- | --- | --- | --- |
| Make referrals | 81% | 51% | 79% |
| Clinical decision making | 79% | 51% | 75% |
| Discharge planning | 85% | 47% | 83% |
| Population health analytics | 67% | 45% | 64% |
| Community needs assessment | 67% | 43% | 62% |
| Support screening conducted internally | 36% | 36% | 0% |

Table A4: Relationship between source(s) and use of HRSN data received from other hospitals/health systems

| Panel A: | (1) Support screening | | (2) Make referrals | | (3) Population health | |
| --- | --- | --- | --- | --- | --- | --- |
| VARIABLES | AME | (95% CI) | AME | (95% CI) | AME | (95% CI) |
| Sources of HRSN Data |  |  |  |  |  |  |
| HIEs | 0.178** | (0.123 - 0.234) | 0.066** | (0.016 - 0.116) | 0.212** | (0.162 - 0.263) |
| EHR vendor network | -0.015 | (-0.068 - 0.039) | 0.083** | (0.037 - 0.130) | 0.148** | (0.099 - 0.198) |
| National network | 0.177** | (0.123 - 0.231) | 0.045+ | (-0.002 - 0.092) | 0.049* | (0.000 - 0.098) |
| SS/CBRP | 0.134** | (0.082 - 0.186) | 0.061** | (0.015 - 0.108) | 0.085** | (0.038 - 0.132) |
| Other HCOs | 0.109** | (0.047 - 0.171) | 0.160** | (0.111 - 0.208) | 0.114** | (0.060 - 0.169) |
| C/SS orgs | -0.074* | (-0.135 - -0.014) | 0.092** | (0.040 - 0.144) | 0.067* | (0.014 - 0.120) |
| Bed size (ref: Small) |  |  |  |  |  |  |
| Medium | -0.002 | (-0.067 - 0.063) | 0.086** | (0.030 - 0.142) | 0.080** | (0.023 - 0.136) |
| Large | 0.052 | (-0.030 - 0.133) | 0.095** | (0.027 - 0.162) | 0.107** | (0.034 - 0.180) |
| Rural | -0.018 | (-0.097 - 0.062) | -0.064+ | (-0.136 - 0.009) | -0.017 | (-0.090 - 0.056) |
| Region (ref: Northeast) |  |  |  |  |  |  |
| Midwest | -0.105* | (-0.190 - -0.020) | -0.050 | (-0.134 - 0.033) | 0.043 | (-0.035 - 0.121) |
| South | -0.256** | (-0.334 - -0.179) | -0.030 | (-0.111 - 0.051) | 0.001 | (-0.078 - 0.079) |
| West | -0.190** | (-0.277 - -0.104) | -0.116* | (-0.211 - -0.020) | -0.023 | (-0.109 - 0.063) |
| Critical access hospital | -0.013 | (-0.092 - 0.066) | -0.004 | (-0.072 - 0.065) | 0.026 | (-0.043 - 0.095) |
| System-affiliated | -0.032 | (-0.102 - 0.039) | 0.092** | (0.031 - 0.153) | 0.151** | (0.087 - 0.215) |
| Ownership (ref: For-profit) |  |  |  |  |  |  |
| Non-profit | -0.173** | (-0.277 - -0.069) | 0.051 | (-0.047 - 0.148) | 0.022 | (-0.074 - 0.119) |
| Government | -0.214** | (-0.340 - -0.088) | 0.060 | (-0.037 - 0.158) | -0.041 | (-0.155 - 0.074) |
| Value-based care | -0.071* | (-0.127 - -0.015) | -0.031 | (-0.078 - 0.016) | -0.034 | (-0.082 - 0.014) |
| Safety-net hospital | 0.047+ | (-0.008 - 0.102) | 0.052* | (0.004 - 0.101) | -0.024 | (-0.076 - 0.028) |
| Observations | 1,562 |  | 1,562 |  | 1,562 |  |

| Panel B | (4) Community needs assessment | | (5) Clinical decision-making | | (6) Discharge planning | |
| --- | --- | --- | --- | --- | --- | --- |
| VARIABLES | AME | (95% CI) | AME | (95% CI) | AME | (95% CI) |
| Sources of HRSN Data |  |  |  |  |  |  |
| HIEs | 0.145** | (0.091 - 0.199) | 0.139** | (0.085 - 0.193) | 0.028 | (-0.025 - 0.080) |
| EHR vendor network | 0.227** | (0.175 - 0.279) | -0.031 | (-0.081 - 0.018) | 0.004 | (-0.048 - 0.055) |
| National network | 0.023 | (-0.028 - 0.074) | 0.036 | (-0.013 - 0.085) | 0.077** | (0.026 - 0.127) |
| SS/CBRP | 0.040 | (-0.009 - 0.089) | 0.079** | (0.031 - 0.128) | 0.057* | (0.007 - 0.108) |
| Other HCOs | 0.121** | (0.064 - 0.177) | 0.154** | (0.104 - 0.204) | 0.196** | (0.145 - 0.247) |
| C/SS orgs | 0.083** | (0.027 - 0.139) | 0.103** | (0.050 - 0.156) | 0.096** | (0.038 - 0.155) |
| Bed size (ref: Small) |  |  |  |  |  |  |
| Medium | -0.003 | (-0.067 - 0.060) | 0.008 | (-0.053 - 0.070) | 0.039 | (-0.023 - 0.102) |
| Large | 0.003 | (-0.081 - 0.087) | -0.001 | (-0.082 - 0.081) | 0.071+ | (-0.008 - 0.150) |
| Rural | -0.030 | (-0.106 - 0.045) | -0.083* | (-0.159 - -0.006) | -0.071+ | (-0.148 - 0.007) |
| Region (ref: Northeast) |  |  |  |  |  |  |
| Midwest | 0.079* | (0.003 - 0.154) | -0.052 | (-0.136 - 0.032) | 0.049 | (-0.035 - 0.133) |
| South | 0.062 | (-0.014 - 0.138) | -0.035 | (-0.116 - 0.046) | 0.058 | (-0.023 - 0.139) |
| West | -0.006 | (-0.090 - 0.078) | -0.003 | (-0.093 - 0.088) | -0.002 | (-0.096 - 0.091) |
| Critical access hospital | -0.066+ | (-0.144 - 0.011) | -0.021 | (-0.096 - 0.053) | -0.008 | (-0.084 - 0.068) |
| System-affiliated | 0.104** | (0.034 - 0.173) | 0.057+ | (-0.009 - 0.124) | 0.029 | (-0.038 - 0.097) |
| Ownership (ref: For-profit) |  |  |  |  |  |  |
| Non-profit | 0.026 | (-0.089 - 0.140) | 0.046 | (-0.058 - 0.149) | 0.058 | (-0.050 - 0.166) |
| Government | -0.006 | (-0.135 - 0.124) | 0.092+ | (-0.008 - 0.193) | 0.082 | (-0.027 - 0.190) |
| Value-based care | -0.012 | (-0.064 - 0.039) | 0.004 | (-0.046 - 0.053) | 0.057* | (0.003 - 0.111) |
| Safety-net hospital | -0.006 | (-0.059 - 0.048) | 0.018 | (-0.032 - 0.067) | 0.045+ | (-0.006 - 0.097) |
| Observations | 1,562 |  | 1,562 |  | 1,562 |  |

Notes: Each column reports output from a separate weighted logistic regression. All coefficients are reported in terms of average marginal effects (AME). 95% Confidence Intervals (CI) are in parentheses. Acronyms used for sources of HRSN data include: HIEs=Health Information Exchanges, EHR=Electronic Health Record, SS/CBRP=Social service or community-based referral platform, Other HCOs=Other health care organizations, C/SS orgs=Community/social service organizations. Asterisks indicate statistically significant difference from reference category: **P<.01, *P < .05. + Indicates marginal significance: P<.10.
